# Supplementary material for: Identification of Molecular Subtypes and Prognostic Characteristics of Adrenocortical Carcinoma Based on Unsupervised Clustering
Source: Int J Mol Sci. 2023 Oct 23;24(20):15465. doi: 10.3390/ijms242015465 (PMC10607826; doi:10.3390/ijms242015465)
Supplement: Supplementary file 1 [file ijms-24-15465-s001.zip › Supplementary Figures.pdf]

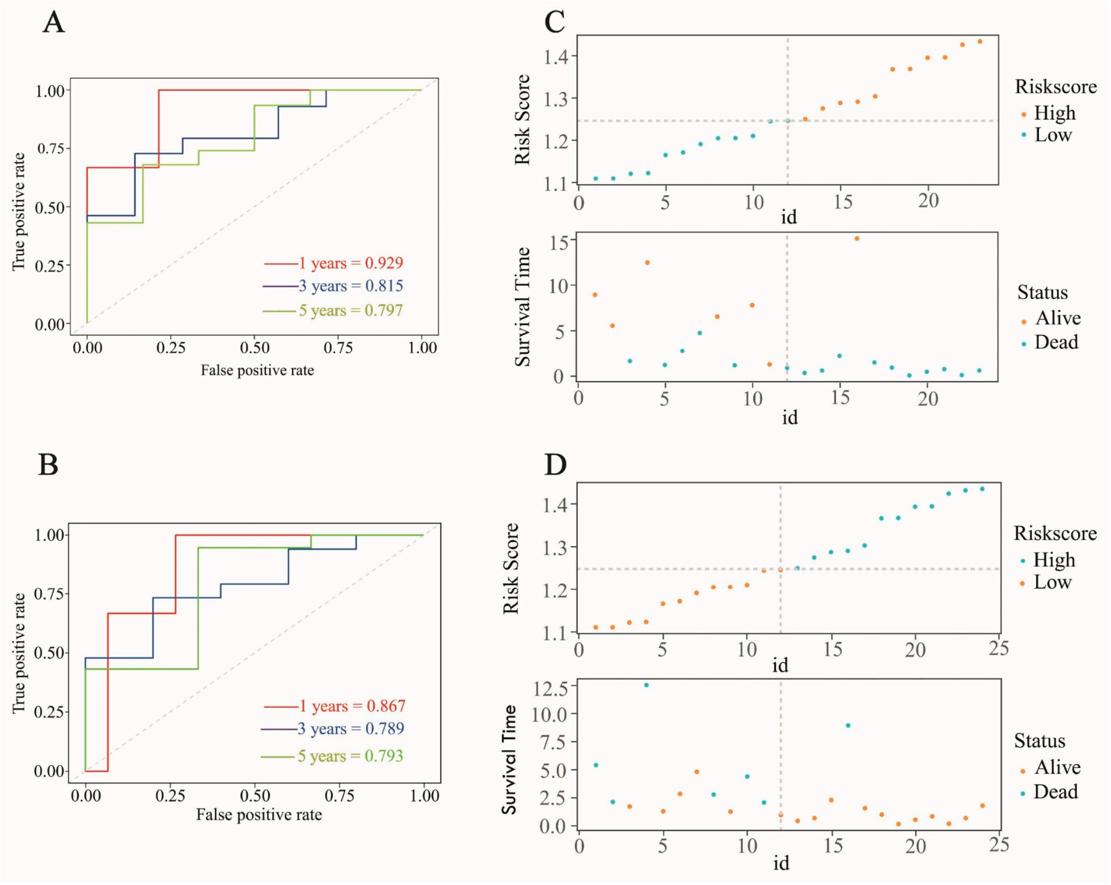

Figure S1. Prognostic characteristics of IRGS in GEO cohort. (**A)** Time-ROC curve to predict the OS at year 1, 3, and 5 in GSE33371 dataset. (**B**) Time-ROC curve to predict the OS at year 1, 3, and 5 in GSE10927 dataset. (**C**) The distribution of risk score and survival status in GSE33371 dataset. (**D**) The distribution of risk score and survival status GSE10927 dataset.

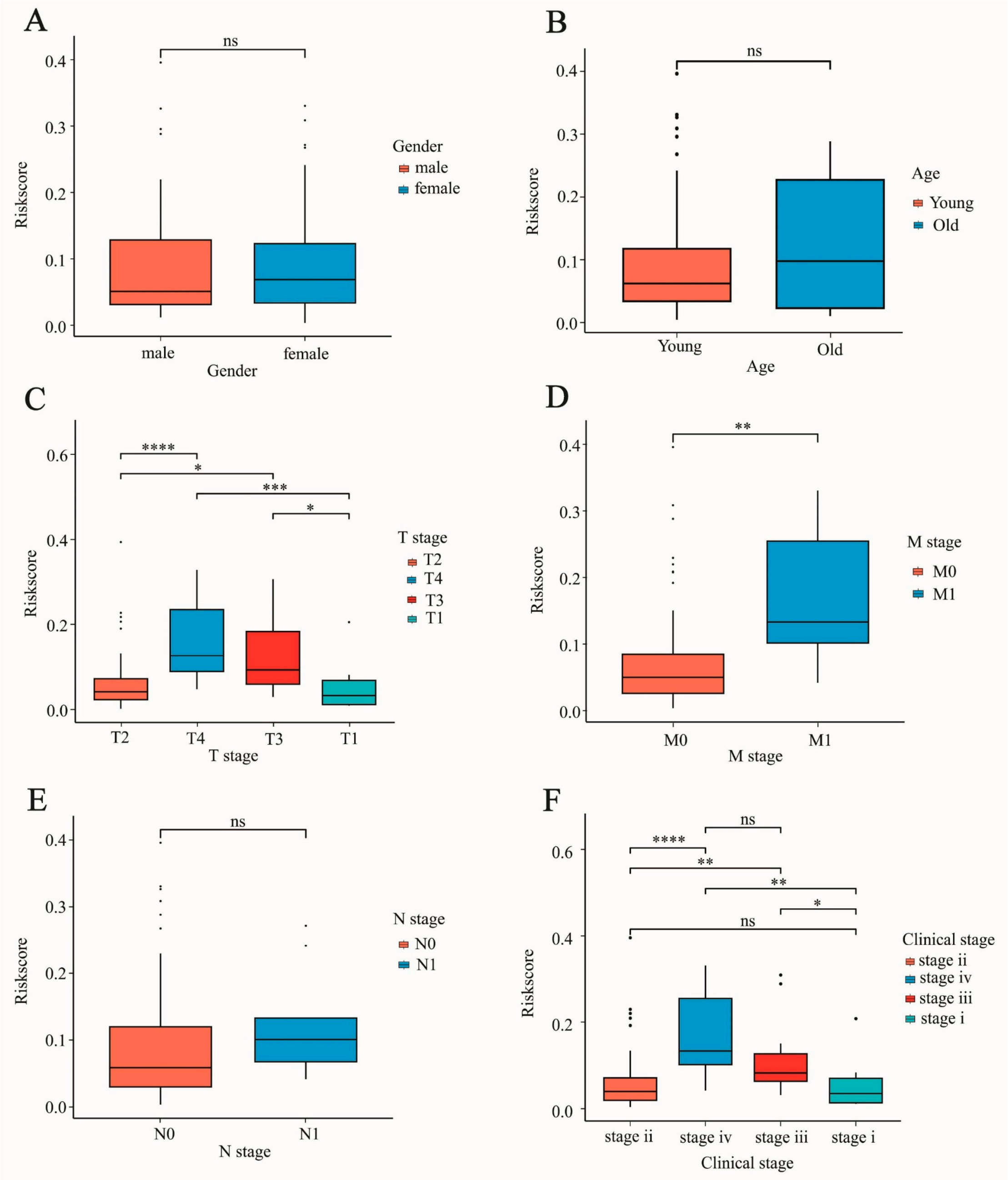

Figure S2. The relationship between clinicopathological features and risk scores in TCGA-ACC patients. (**A**–**F**) Comparison of risk scores in patients with different genders, ages, T stages, M stages, N stages and clinical stages. Data in (**A**–**F**) were analyzed by Wilcoxon test; ns, no significance, * $p < 0.05$, ** $p < 0.01$, *** $p < 0.001$ and **** $p < 0.0001$.

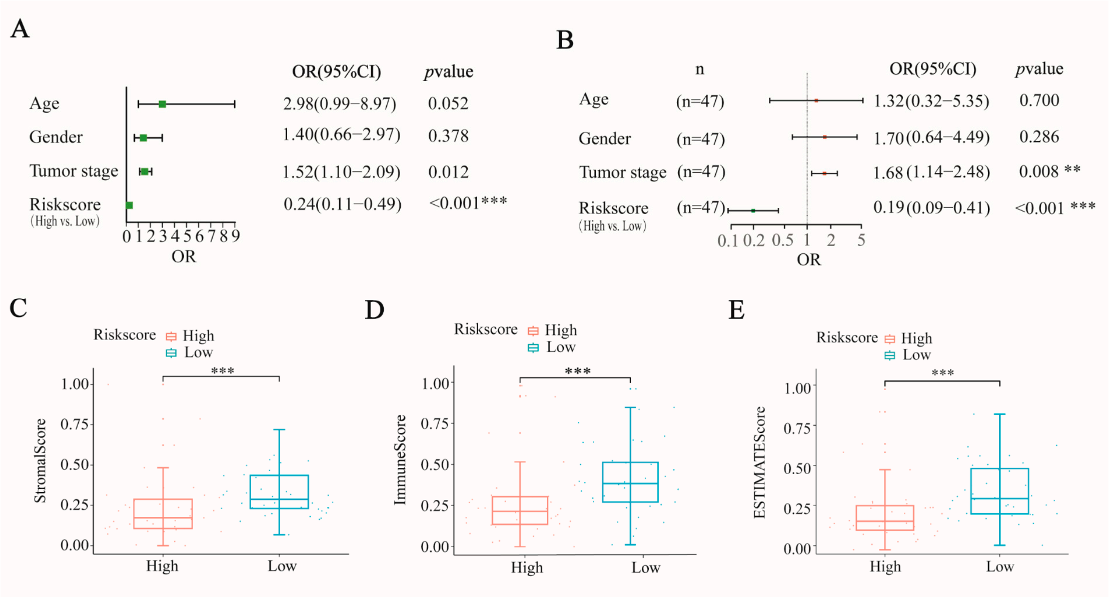

Figure S3. The results of univariate and multivariate Cox analyses in GEO cohort. (**A**) the forest plot of univariate Cox regression analysis in GEO cohort. The green color block indicates that OR＞0 in the univariate Cox analysis. (**B**) the forest plot of multivariate Cox regression analysis in GEO cohort. The green color block indicates OR < 1, the red color block indicates OR > 1 (**C**) The correlation analysis between stromal scores and risk scores via "ESTIMATE" algorithm. (**D**) The correlation analysis between immune scores and risk scores via "ESTIMATE" algorithm. (**E**) The correlation analysis between estimate scores and m7G-related scores via "ESTIMATE" algorithm. Data in (**C-E**) were analyzed by Wilcoxon test; ** $p < 0.01$ and *** $p < 0.001$.
